# Subacute poisoning with bifenthrin increases the level of interleukin 1ß in mice kidneys and livers

**DOI:** 10.1186/s40360-021-00490-1

**Published:** 2021-04-26

**Authors:** Oktawia Pylak-Piwko, Barbara Nieradko-Iwanicka

**Affiliations:** 1 Center of Oncology of the Lublin Region St. John from Dukla, Jaczewski Street 7, 20-090 Lublin, Poland; 2 Chair and Department of Hygiene, Medical University of Lublin, Radziwillowska Street 11, 20-080 Lublin, Poland

**Keywords:** Bifenthrin, interleukin1ß, Pro-inflammatory properties

## Abstract

**Background:**

Bifenthrin is a pyrethroid. Chronic exposure of humans to the pesticide occurs. Reports about immunotoxicity and proinflammatory effect of pyrethroids were published. The aim of the article was to check if subacute poisoning with bifenthrin affects proinflammatory interleukin 1ß and tumor necrosis factorα (TNFα) in kidneys, livers and the function of these organs.

**Methods:**

Thirty two female mice were used. They were divided into 4 groups: controls, mice receiving 1.61 mg/kg bifenthrin for 28 days (group 1), 4.025 mg/kg (2), 8.05 mg/kg (3). On day 29 they were sacrificed, blood, livers and kidneys were obtained. Creatinine concentration and alanine transaminase (ALT) activity were estimated in the blood sera. Interleukin1ß and TNFα concentrations in the organs were measured.

**Result:**

Mean interleukin 1ß concentration in the livers of controls was 53 pg/ml, in group 1- 54 pg/ml, 2- 59 pg/ml, 3- 99 pg/ml (*p* < 0.05 vs controls). It was accompanied by significant increase in ALT activity in group 3 vs controls (*p* < 0.05). In the control kidneys interleukin 1ß was 3.9 pg/ml, group 1–6.8 pg/ml, 2–9.8 pg/ml and 3- 11 pg/ml. Statistically significant difference between group 1, 2 and 3 vs controls was found. There was no significant differences among the groups in TNFα concentrations neither in the livers nor kidneys.

**Conclusion:**

Subacute poisoning with bifenthrin significantly increases interleukin 1ß concentration in livers and kidneys in a dose-proportionate level. It is accompanied by ALT activity increase. It confirms nephrotoxic and hepatotoxic and pro-inflammatory effect of bifenthrin in non-target organisms.

## Background

Bifenthrin belongs to type I pyrethroid insecticides interacting with voltage-gated sodium channels in neuron membranes [1–3]. It is neurotoxic. Intoxication leads to death in target organisms. It was believed that pyrethroids were safe for mammals as they were metabolized in the liver (cleaved at the central ester bond). The metabolites: cis-3-(2-chloro-3,3,3-trifluoroprop-1-en-1-yl)-2,2-dimethylcyclopropane carboxylic acid (CFMP) and 3-phenoxybenzoic acid (3-PBA) are considered relatively non-toxic and are passed with urine. The studies conducted by Wielgomas et al. showed that pyrethroid metabolites were detected in urine samples of urban and rural populations [4, 5]. There is evidence that pyrethroid intoxication in mammals (humans and animals) may lead to health problems [6–8]. Acute poisoning with bifenthrin in mammals produces aggressive sparring, sensitivity to stimuli and tremor [1, 9, 10]. As bifenthrin is used in agriculture and horticulture for pest control, there is a risk of chronic exposure of humans with pesticide residues in food products. The compound is considered moderately toxic in vivo for vertebrates if compared with other pyrethroids [11]. There are recent reports about possible immunotoxicity and proinflammatory effect of pyrethroids in vertebrates developing due to oxidative stress. Glutathione-S transferases (GSTs) have a function in bifenthrin metabolism. Bifenthrin and other pyrethroids inhibit GSTs in a competitive mechanism in the livers of non-target organisms producing oxidative stress [12–15].

The aim of the study was to check if subacute poisoning with bifenthrin affects proinflammatory interleukin 1ß and tumor necrosis factorα (TNFα) in kidneys as well as livers and the function of these organs.

## Methods

### Settings

The study project was approved by The Local Ethical Committee in Lublin, Poland and Institutional Animal Care and Use Committee at the Medical University of Lublin, Poland. Both authors had a training in planning and conducting experiments on animals. Bifenthrin was purchased form Organic Chemistry Institute (Annopol, Warsaw, Poland). Saline was purchased from Glenmark Pharmaceuticals (Warsaw, Poland).

The experiment was conducted at the Centre for Experimental Medicine at The Medical University of Lublin. The European, Polish and issued by the Medical University of Lublin guidelines for using animals were followed. There were standard laboratory conditions (12 h light/12 h dark cycle, temperature 21–22 °C, air humidity 55–60%). The animals were bred at the Centre for Experimental Medicine at The Medical University of Lublin (breeder No 077 registered at the Ministry of Science and Higher Education, Warsaw, Poland). The herd originated from Charles River (Cologne, Germany). The mice had free access to water (sterilized with UV) and animal feed ad libitum. The feed for rodents was purchased from Altromin International (Lage, Germany).

### Sample size

A total of 32 of 6-week-old (they were young non-pregnant adults) female Albino Swiss mice weighing 20–26 g at the beginning of the study were used. They were randomly divided into 4 groups of 8:

- controls receiving saline daily *i.p*. for 28 days
- animals receiving bifenthrin *i.p*. at the dose of 1.61 mg/kg for 28 days (group 1)
- animals receiving bifenthrin *i.p*. at the dose of 4.025 mg/kg for 28 days (group 2)
- animals receiving bifenthrin *i.p*. at the dose of 8.05 mg/kg for 28 days (group 3).

The doses were chosen according to our previous experience with this pyrethroid [15]. The dose of 8.05 mg/kg was considered 0.5 LD_50_ [15].

### Tests and measurements

On day 29 the animals were sacrificed by decapitation. There was no anaesthesia before as we were concerned about biochemical blood parameters. Samples of venous blood were obtained. The livers and kidneys were weighted with a laboratory weighing scales (MFD manufactured by A&D CO., LTD, Seoul, Korea). The organs were homogenized with a mechanical blender MPW-120 (MPW Med. Instruments, Warsaw, Poland) in 0.1 mol buffer of Tris-HCl, of pH 7.4. The sample of 0.5 g of tissue was blended in 5 ml of buffer. The homogenates were centrifuged for 15 min (5000×g) twice. The Sigma1-6P centrifuge (Polygen, Engelwood, NY, USA) was used. The supernatant was used for measuring IL-1ß and TNFα concentrations with ELISA tests. The IL-1ß and TNFα ELISA kits were purchased from the manufacturer (Cloud-Clone Corp., Houston, TX, USA). Creatinine concentration and alanine transaminase (ALT) activity in the blood sera were measured with ErbaMannheim XL-600 biochemistry analyzer (Mannheim, Germany).

### Statistical analysis

The results were analysed with IBM SPSS Statistics (v. 21) (Statsoft Sp.zo.o., Cracow, Poland). The compliance of the distribution of variables with the hypothetical normal distribution was checked with the Shapiro-Wilk normality test. The obtained results were presented using the basic elements of descriptive statistics (mean ± SD as the results were normally distributed).

The comparative analysis for the studied variables was carried out with the use of parametric statistical tests. For quantitative features of continuous character with normal distributions, the parametric Student’s t-test was used, comparing the results from two groups. In the remaining cases, the Mann-Whitney U was used for two samples for quantitative traits. The observed differences were considered statistically significant at *p* < 0.05.

## Results

Kidney mass did not differ among the groups. It was (Mean ± SD) 0.16 ± 0.02 g in controls, 0.16 ± 0.01 g in group 1, 0.16 ± 0.03 g in group 2 and 0.16 ± 0.04 g in group 3. Neither did the liver mass as it was 1.24 ± 0.18 g in the controls, 1.33 ± 0.16 g in group 1, 1.35 ± 0.2 g in group 2, 1.35 ± 0.2 g in group 3. Creatinine concentration did not differ among the groups and it was 0.2 ± 0.0 mg/dl in control group, 0.2 ± 0.02 mg/dl in group 1, 0.2 ± 0.03 mg/dl in group 2 and 0.2 ± 0.04 mg/dl in group 3. The ALT activity was 51 ± 12 U/l in controls, 60 ± 13 U/l in group 1, 80 ± 9 U/l in group 2 and 135 ± 20 U/l in group 3. The difference between group 3 and controls was statistically significant (*p* < 0.05). The interleukin 1ß concentration in the livers of controls was 53.5 ± 23.9 pg/ml, in group 1–54.1 ± 33.5 pg/ml, 2–59.1 ± 60.0 pg/ml (*p* < 0.05), 3–99 ± 79.9 pg/ml (*p* < 0.05 vs controls). In the kidneys of controls it was 3.9 ± 2.3 pg/ml, group 1–6.8 ± 10.6 pg/ml, 2–9.8 ± 7.9 pg/ml and 3–11.2 ± 5.2 pg/ml. There was a statistically significant difference between group 1,2 and 3 vs controls (*p* < 0.05). The TNFα levels did not significantly differ in the groups exposed to bifenthrin in comparison with controls neither in the livers nor in the kidneys. The liver damage biomarkers were shown it Table 1 and kidney biomarkers in Table 2.

**Table 1:** The influence of bifenthrin on liver mass, serum ALT activity, interleukin 1ß and TNFα in mice livers

Group	Liver mass [g] (mean ± SD)	ALT [U/l] (mean ± SD)	Interleukin 1ß in the livers [pg/ml] (mean ± SD)	TNFα in the livers [pg/ml] (mean ± SD
Control females (*n* = 8)	1.24 ± 0.18	51 ± 12	53.5 ± 23.9	5.81 ± 1.5
1 (*n* = 8)	1.33 ± 0.16	60 ± 13	54.1 ± 33.5	6.39 ± 1.2
2 (*n* = 8)	1.35 ± 0.20	80 ± 90	59.1 ± 60.0	7.62 ± 2.4
3 (*n* = 8)	1.35 ± 0.20	135 ± 20*	99 ± 79.9*	7.90 ± 5.2

*p < 0.05 vs controls

Group 1 - females receiving bifenthrin *i.p*. at the dose of 1.61 mg/kg for 28 days

Group 2 - females receiving bifenthrin *i.p*. at the dose of 4.025 mg/kg for 28 days

Group 3 - receiving bifenthrin *i.p*. at the dose of 8.05 mg/kg for 28 days

**Table 2:** The influence of bifenthrin on kidney mass, creatinine serum concentration, interleukin 1ß and TNFα in mice kidneys

Group	Kidney mass [g] (mean ± SD)	Creatinine [mg/dl] (mean ± SD)	Interleukin 1ß in the kidneys [pg/ml] (mean ± SD)	TNFα in the kidneys [pg/ml] (mean ± SD)
Control females (*n* = 8)	0.16 ± 0.02	0.2 ± 0.00	3.9 ± 2.3	5.45 ± 1.1
1 (*n* = 8)	0.16 ± 0.01	0.2 ± 0.02	6.8 ± 10.6*	6.04 ± 1.6
2 (*n* = 8)	0.16 ± 0.03	0.2 ± 0.03	9.8 ± 7.9*	6.39 ± 2.1
3 (*n* = 8)	0.16 ± 0.04	0.2 ± 0.04	11.2 ± 5.2*	8.50 ± 3.6

*p < 0.05 vs controls

Group 1 - females receiving bifenthrin *i.p*. at the dose of 1.61 mg/kg for 28 days

Group 2 - females receiving bifenthrin *i.p*. at the dose of 4.025 mg/kg for 28 days

Group 3 - receiving bifenthrin *i.p*. at the dose of 8.05 mg/kg for 28 days

## Discussion

In our experiment it was shown that there was a significant increase of interleukin 1ß in mice livers and kidneys. It was proportionate to dose of bifenthrin used in the experimental model of subacute poisoning. The kidneys were more affected by the xenobiotic than livers. In the study of Pawar et al. it was explained that intoxication with pyrethroids produced oxidative stress in the kidneys. It was confirmed by detecting a significant decrease in the levels of thiols in the kidneys of animals exposed to a pyrethroid. Intoxication with the xenobiotic caused a significant decrease in superoxide dismuthase (SOD) and catalase activity in the kidneys. Histopathological evaluation of the kidneys revealed hemorrhages in the cortex and core, tubular degenerative changes with closure of the lumen and reduction of Bowman’s capsule space [16]. In our study, due to limited financial resources we couldn’t perform histopathological examination of internal organs. The hepatotoxic effect of bifenthrin was visible in our study as we detected an increased activity of ALT in the group exposed to the highest dose of the pesticide and significant increase of interleukin 1ß concentration in the group. The IL-1β and TNFα are synthesized in macrophages and monocytes. They are secreted into the blood, thanks to which they have a systemic effect. TNFα is one of the first cytokines to appear during inflammation [17]. TNFα stimulates increasing production of interleukin 1ß, which happens during oxidative stress lasting for longer time [18]. This may explain why in the course of our experiment lasting for 28 days TNFα did not significantly differ among the experimental groups, and interleukin 1ß did. In another study conducted at our department it was shown that subacute poisoning with another pyrethroid lambdacyhalothrin also produced a significant increase of interleukin 1ß in mice kidneys and livers [19].

In a similar study with bifenthrin it was used at the dose of 8 mg/kg and decreased locomotor activity in mice confirming its’ neurotoxic effect. Moreover it increased ALT activity in mice blood sera and led to formation of lymphocyte infiltrations in the livers after subacute poisoning showing that apart from neurons, poisoning with bifenthrin produces liver malfunctioning too [15]. In the present study we could not perform histopathologic examination, but learning from the cited publication, we expected similar changes in the livers.

The liver is very likely to get damaged by all xenobiotics that are metabolized in that organ. Bifenthrin undergoes oxidative metabolism leading to the formation of 4′-hydroxy-bifenthrin and hydrolysis hepatic microsomes in rodents as well as in humans [20, 21]. In our study we used young adult mice, as young rodents have lower ability to metabolize pyrethroids. In rodent pups exposed to bifenthrin, the pesticide can cross their blood-brain barrier, accumulate in the brain and produce log lasting neurobehavioral deficits [22]. Dar et al. administered bifenthrin to rats for up to 30 days induced an oxidative stress in the livers, kidneys and lungs [23].

There are publications about the immunotoxic effects of bifenthrin on zebrafish embryos. In the experiment conducted by Jin et al. it was shown that exposure to bifenthrin increased the level of interleukin 1ß, interleukin 8, caspase 9 and 3 in embryos exposed to S-cis- bifenthrin [14]. Park et al. confirmed that bifenthrin intoxication during zebrafish embryogenesis induced developmental toxicity, inflammation and decreases angiogenesis [20].

Interleukin 1 is a signalling molecule that regulates immune response, mediates functioning of leukocytes, lymphocytes, acts as a pyrogen and mediates development of chronic inflammation [24]. Therefore it was chosen in our experiment as a marker of tissue stress and inflammatory response to the xenobiotic. TNFα is also a proinflammatory cytokine playing a role in autoimmune disorders and neoplasm development.

The results of our study are similar to the results obtained by Wang et al. who conducted an experiment on male mice exposed to bifenthrin for 3 weeks and confirmed immunotoxic effect of the pyrethroid [25]. Another study published by Wang et al. elucidated the mechanisms of bifenthrin’s immunotoxicity in murine macrophages. Exposure to bifenthrin inhibited transcription levels of interleukin 6 and TNFα due to lipopolysaccharide stimulation. Intoxication with bifenthrin increased reactive oxygen species and led to oxidative stress related genes’ dysregulation [26].

In a recent publication Jin et al. confirmed that oral exposure of male mice to bifenthrin at the dose of 20 mg/kg for 3 weeks increased the levels of proinflammatory cytokines and reduced activities of antioxidant enzymes (SOD, glutathione peroxidase) due to oxidative stress in the course of poisoning with the pyrethroid [27]. Many other authors agree that pyrethroids damage internal organs via oxidative stress [28–30].

There is also data about immunomodulating effect of pyrethroids in humans. In the study conducted by Neta et al. numerous proinflammatory cytokines were measured in blood samples obtained from umbilical cord at birth from 300 newborn children (including interleukin 1ß, TNFα, interleukin 6, interleukin 10) with prenatal exposure to permethrin. Authors found a decrease of interleukin 10 in those children which might increase the risk for allergic diseases and asthma later in life [31]. Meanwhile permethrin, like bifentrin belonging to type I pyrethroids, is recommended for malaria prevention by the World Health Organisation even for pregnant women [32]. Surely the benefit of using it must overweigh the possible side effects.

Our study shows that even low dose of bifenthrin causes a significant increase interleukin 1ß in mice kidney which demonstrates that inflammation in the kidney occurs even at very low doses. It is connected with the fact that metabolites of the pyrethroid are eliminated with urine. The 3-PBA is the most commonly detected urinary metabolite of several pyrethroids [33]. It is detected in urine of children and adults from rural and urban areas, confirming widespread exposure of human population to these compounds [4]. Studies in animal models support this statement. Amin et al. confirmed nephrotoxicity of deltamethrin, in catfish [34]. Abdel-Daim et al. also recorded pyrethroid’s nephrotoxic effect in tilapia [35].

Although modern laboratories are able to measure different immunotoxicity, hepatotoxicity and nephrotoxicity biomarkers, due to limited financial resources we selected only the few ones shown above.

## Conclusions

Subacute poisoning with bifenthrin significantly increases interleukin 1ß concentration in livers and kidneys in a dose-proportionate level. It is accompanied by ALT increase. It confirms nephrotoxic and hepatotoxic and pro-inflammatory effect of bifenthrin in non-target organisms.

## Data Availability

All data and materials are available at the corresponding author.

## Abbreviations

ALT: Alanine transaminase
i.p.: Intraperitoneally
LD_50_: Lethal dose for 50% of the exposed population
SD: Standard deviation
SOD: Superoxide dismuthase
TNFα: Tumor necrosis factor α
